# ‘We had to manage what we had on hand, in whatever way we could’: adaptive responses in policy for decentralized drug-resistant tuberculosis care in South Africa

**DOI:** 10.1093/heapol/czaa147

**Published:** 2021-02-13

**Authors:** Karina Kielmann, Lindy Dickson-Hall, Waasila Jassat, Sacha Le Roux, Mosa Moshabela, Helen Cox, Alison D Grant, Marian Loveday, Jeremy Hill, Mark P Nicol, Koleka Mlisana, John Black

**Affiliations:** Institute of Global Health and Development, Queen Margaret University, Edinburgh EH21 6UU, UK; Division of Medical Microbiology, Faculty of Medicine, University of Cape Town, South Africa; School of Public Health, University of the Western Cape; Division of Medical Microbiology, Faculty of Medicine, University of Cape Town, South Africa; Africa Health Research Institute, School of Nursing and Public Health, University of KwaZulu-Natal, South Africa; Institute for Infectious Disease and Molecular Medicine and Wellcome Centre for Infectious Disease Research in Africa, University of Cape Town, Cape Town, South Africa; Africa Health Research Institute, School of Nursing and Public Health, University of KwaZulu-Natal, South Africa; London School of Hygiene & Tropical Medicine, TB Centre, UK; School of Public Health, University of the Witwatersrand, South Africa; Health Systems Research Unit, South African Medical Research Council; Division of Medical Microbiology, Faculty of Medicine, University of Cape Town, South Africa; London School of Hygiene & Tropical Medicine, TB Centre, UK; Division of Medical Microbiology, Faculty of Medicine, University of Cape Town, South Africa; Infection and Immunity, School of Biomedical Sciences, Faculty of Health and Medical Sciences, University of Western Australia, Perth, Australia; Department of Medical Microbiology, University of KwaZulu-Natal, Durban, South Africa; Department of Infectious Diseases, Livingstone Hospital, Lindsay Rd, Industrial, Port Elizabeth, 6020, South Africa

**Keywords:** Policy implementation, decentralization, drug-resistant tuberculosis, health systems, readiness

## Abstract

In 2011, the South African National TB Programme launched a policy of decentralized management of drug-resistant tuberculosis (DR-TB) in order to expand the capacity of facilities to treat patients with DR-TB, minimize delays to access care and improve patient outcomes. This policy directive was implemented to varying degrees within a rapidly evolving diagnostic and treatment landscape for DR-TB, placing new demands on already-stressed health systems. The variable readiness of district-level systems to implement the policy prompted questions not only about differences in health systems resources but also front-line actors’ capacity to implement change in resource-constrained facilities. Using a grounded theory approach, we analysed data from in-depth interviews and small group discussions conducted between 2016 and 2018 with managers (*n* = 9), co-ordinators (*n* = 15), doctors (*n* = 7) and nurses (*n* = 18) providing DR-TB care. Data were collected over two phases in district-level decentralized sites of three South African provinces. While health systems readiness assessments conventionally map the availability of ‘hardware’, i.e. resources and skills to deliver an intervention, a notable absence of systems ‘hardware’ meant that systems ‘software’, i.e. health care workers (HCWs) agency, behaviours and interactions provided the basis of locally relevant strategies for decentralized DR-TB care. ‘Software readiness’ was manifest in four areas of DR-TB care: re-organization of service delivery, redressal of resource shortages, creation of treatment adherence support systems and extension of care parameters for vulnerable patients. These strategies demonstrate adaptive capacity and everyday resilience among HCW to withstand the demands of policy change and innovation in stressed systems. Our work suggests that a useful extension of health systems ‘readiness’ assessments would include definition and evaluation of HCW ‘software’ and adaptive capacities in the face of systems hardware gaps.

**KEY MESSAGES**

- Health system readiness assessments highlight critical resource gaps but fail to capture local contextual factors and values and the dynamic responses of health systems actors in driving policy implementation.
- Different levels of readiness to decentralize drug-resistant tuberculosis care observed in South African districts can be partly explained by health systems actors’ capacity to adapt to ongoing challenges and new demands placed on a stressed health system.
- Health systems’ actors’ capacities to adapt, self-organize and devise locally relevant strategies to implement policy directives reflect dimensions of *resilience* to systems stressors as well as *readiness* for organizational change.
- Conventional readiness assessment tools could be usefully extended to include questions about how health workers and managers respond to both the ‘everyday’ crises and the sporadic policy changes that can disrupt service delivery.

This policy is in place, but we don’t know how to implement it… so let’s work with the district, because they had it on paper, it was there… but we actually had to modify the [National Department of Health] policy (*Senior clinician, KwaZulu-Natal*).

## Introduction

In 2006, medical journals worldwide reported the outbreak of extensively drug-resistant tuberculosis (XDR-TB) in a hospital in Tugela Ferry, in the province of KwaZulu-Natal (KZN), South Africa. Tugela Ferry was a critical ‘tipping point’ for drug-resistant (DR)-TB in the country and drew global attention to the threat of DR-TB (Saidi *et al.*, 2017). However, despite recognition that the conventional model of institutionalized treatment was inadequate (Padayatchi and Friedland, 2008), programmatic response to DR-TB was variable and fairly sluggish in some provinces (WHO, 2016). The numbers of DR-TB patients rose from ∼8000 between 2007 and 2010 to >16 000 in 2017 (WHO, 2018).

Five years after the outbreak, the South African National TB Programme launched a policy of ‘decentralized and deinstitutionalized’ management of DR-TB (Department of Health and Republic of South Africa, 2011a,b) to expand facilities’ capacity to manage DR-TB. Concurrently, the diagnostic and treatment landscape for DR-TB was rapidly evolving^1^ and placing new demands on an already-stressed health system. Significant gaps between the number of people found to have DR-TB and those starting on second-line treatment (Cox *et al.*, 2017a; Evans *et al.*, 2018) prompted questions about the health system’s capacity to provide timely and appropriate treatment at decentralized levels of the system.

In South Africa, large regional disparities in disease burden, human resources, financing and investment, administration and management capacity are mirrored in considerable differences in service readiness and availability (Dookie and Singh, 2012; Fusheini and Eyles, 2016). By 2015, substantially different ‘models’ of decentralized care for DR-TB were emerging, reflecting not only different interpretations of the policy, but variability in district health systems contexts, capacity and readiness to implement decentralized care (Cox *et al.*, 2015; Department of Health, Republic of South Africa, 2019).

Within the literature on policy implementation, ‘readiness’ refers to systems capability to initiate and sustain organizational change in response to initiatives intended to improve systems performance (Manu *et al.*, 2018; Zurovac *et al.*, 2018). Assessments of health systems readiness traditionally involve an evaluation of the minimum ‘hardware’ requirements to ensure successful delivery of health services. Monitoring tools, e.g. the WHO SARA (Service Availability Readiness Assessment) track the functional availability of health system’s ‘building blocks’ components, i.e. ‘…trained staff, guidelines, equipment, diagnostic capacity, and medicines and commodities’ (WHO, 2015). In settings where resources are lacking or inequitably distributed, assessments like the SARA can highlight critical gaps. Yet, they fail to capture contextual factors influencing the supply and demand of health services, the role of values as ‘steering mechanisms’ (Van Olmen *et al.*, 2012), and the dynamic responses of health systems actors in driving or obstructing change (Blaauw *et al.*, 2006).

In this paper, we examine early responses to the mandate to decentralize DR-TB care in three South African provinces to illustrate the dynamic relationship between human agency, health systems readiness and policy implementation. Our aim is to highlight the neglected role of actors’ behaviours and interactions—often referred to as ‘software’ (Sheikh *et al.*, 2011)—in assessments of health systems ‘readiness’. Against the ongoing challenges of providing DR-TB care in resource-constrained facilities, e.g. poorly maintained infrastructure, inadequate drug supplies, overworked staff and insufficient training on DR-TB management, we draw attention to the critical role of adaptive responses in policy implementation.

### Conceptual framework

Calls to more explicitly link policy and systems research (Gilson, 2012; Ghaffar *et al.*, 2016) suggest the need to consider how systems actors respond and adapt to changes that are introduced as a result of new policy initiatives. Policy implementation requires more than a template of standard operating procedures; it is a ‘…challenging process, working through the whole health system and ultimately taking effect or being blocked at the frontlines of service delivery and community engagement’ (Gilson, 2016). To examine the role of human agency in the adoption of health systems interventions, some implementation researchers suggest greater inclusion of concepts used in organizational theory and management (Birken *et al.*, 2017). Here, ‘readiness’ refers to willingness and capacity to implement a particular innovation (Weiner, 2009; Scaccia et al., 2015); the research on organizational readiness explicitly considers interactions between ‘emergent expressions of human agency’ and context as critical to enactment of change within the system (May, 2013).

The concept of ‘tinkering’ further helps to elucidate flexibility and adaptation in local-level responses to policy initiatives. ‘Tinkering’ has been used to describe how actors ‘adjust the protocol to unforeseen events’ (Timmermans and Berg, 1997) through an opportunistic rearrangement of existing elements that opens space for new ways of doing things. Policy translation is both creative and pragmatic, and characterized by ‘fluid multi-actor processes of interpretation, mutation and assemblage. […]’ (Stone, 2017, p. 67). Husain (2017) refers to emergent ‘tinkering’ in decentralized health policymaking in China: in the absence of national standardization and expert support, space became available for local discretion in pragmatic problem-solving, and local context-specific approaches in policy implementation.

Health systems actors’ tinkering can also strengthen the system’s capacity to manage everyday crises faced in resource-constrained settings. An ‘agency centred’ focus of recent thinking on resilience in development work (Jeans *et al.*, 2017) argues that we should ‘….move away from simply looking at what a person, household, or system **has** and recognise and enhance what it **does**’ (sic). Though the concept of resilience commonly refers to systems’ capacity to cope and ‘bounce back’ after significant shocks such as war, natural disasters or humanitarian crises, the term has also been applied to the micro-level adjustments that actors make to address ongoing challenges in constructive ways. For example, Gilson *et al.* (2017) focus on ‘…internally generated chronic stresses, some of which are even infused into the routine organisational life of health systems’ that both generate and demand expressions of ‘everyday resilience’ among district-level managers.

Here, we examine the role of health workers and managers’ adaptive responses to move the agenda on decentralized DR-TB care forward in pragmatic ways, against a backdrop of structural resource constraints, and policy tensions (Moshabela *et al.*, 2020). This involved attention to the small but meaningful changes in normative practice made to adapt to the ‘moving target’ of DR-TB care innovations in the district health system.

## Materials and methods

**Figure 1:**
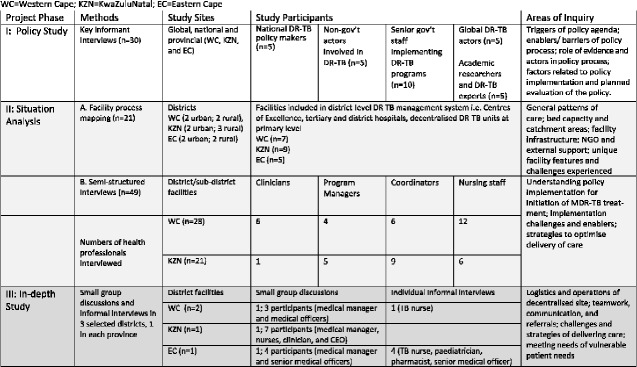
Data collection methods, participants and areas of inquiry. WC, Western Cape; KZN, KwaZulu-Natal; EC, Eastern Cape. Source: Adapted from Department of Health, Republic of South Africa (2019), p. 17.

This paper draws on data from a 4-year project that aimed to gain an understanding of the policy context, patient care pathways and models of decentralization of DR-TB care in three provinces of South Africa: Western Cape (WC); KZN and Eastern Cape (EC). The project entailed three phases of qualitative research conducted between 2016 and 2018 (see Figure 1): a key informant interview (KII) study (Phase 1); facility process-mapping and interviews in sites providing decentralized DR-TB care (Phases 2A and 2B, respectively) and an in-depth study of specific emergent models of decentralized care in the three provinces (Phase 3). We draw mainly on the interviews conducted in Phases 2B and 3, the vast majority of which took place in WC and KZN. We refer to a few of the KII (Phase 1) to elucidate the context within which the policy was launched, more fully described elsewhere (Moshabela *etal.*, 2020).

### Ethical considerations

All research procedures for the project were approved by the Human Research Ethics committee at the University of Cape Town and by the Observational/Interventions Research Ethics Committee of the London School of Hygiene & Tropical Medicine. Permission to conduct the site visits and interviews was granted by the Department of Health research committees in the EC, WC and KZN Provinces. Written informed consent was obtained from all participants.

### Data collection

During Phase 1, KIIs were conducted by co-authors MM and WJ with national and provincial stakeholders (see Figure 1 for details), exploring their understanding of the evidence, timeline and initial strategies for launching the national policy on decentralized DR-TB care. During Phase 2A, site visits were conducted by co-authors LDH, SLR, and a third researcher (LM) in 21 facilities within 13 district-level decentralized sites of care in WC, KZN and EC. Researchers used a structured tool, administered to the facility manager or lead TB nurse, to conduct facility process-mapping while cross-checking pathways of care for patients with rifampicin-resistant TB identified previously using National Health Laboratory Services (NHLS) data, and identify emerging ‘models’ of decentralized care (Hill *et al.*, 2020). In Phase 2B, WJ conducted in-depth interviews in WC and KZN to understand the policy implementation processes, and factors enabling and hindering decentralized DR-TB care across districts. Informants were selected purposively and included those more closely associated with implementation of the decentralization policy.

Permissions for interviews conducted in Phases 1 and 2 were obtained from the respective provinces, district and facility management. While most interviews were conducted face to face, some were done telephonically. After obtaining written informed consent, interviews were voice-recorded; all but one were conducted in English and transcribed by SLR, checked and edited by MM and WJ. One interview was conducted in Afrikaans, translated and transcribed by LDR.

In Phase 3, researchers returned to one district in each province that represented a distinct ‘model’ of decentralized care, as identified in Phase 2A. They conducted small group discussions and informal interviews with staff, mainly at three district ‘hub’ hospitals, to probe contextual features and mechanisms influencing ‘optimal’ delivery of care in the different sites. These conversations were recorded with consent of the participants; one of the researchers (SLR) made detailed notes based on the recordings.

### Data analysis

Adopting a grounded theory approach to analysis (Glaser and Strauss, 1967), researchers (KK, LDH, MM, SLR and WJ) read transcripts and fieldnotes several times to discuss emerging concepts. For this paper, we identified three broad themes: systems readiness, health care worker (HCW) adaptive capacities and local innovations in responding to the mandate to decentralize DR-TB care. Constant comparison of text segments identified as relevant to themes helped to generate more nuanced sub-themes (Appendix 1) that formed the basis of a coding system. The data were manually coded by three researchers (LDH; SLR; KK) in Microsoft Word; relevant quotes were extracted to facilitate comparison and identification of patterns. All individual and health facility identifiers were anonymized, with individuals identified only through their function within the system.

## Results

### The move to decentralize DR-TB care in South Africa: gaps in ‘readiness’

Policy guidelines to manage DR-TB in South Africa were drafted in 2000, and successively revised to emphasize standardized treatment regimens as well as monitoring and surveillance requirements for a uniform approach to organizing DR-TB services. The revised policy framework for decentralized DR-TB care (Department of Health Republic of South Africa, 2019) foresaw the transfer of responsibility for treating DR-TB patients from regional specialist centres to district-level facilities closer to patients’ homes. The policy distinguished between a hospitalized model for patients who were clinically unwell, had second-line resistance, were sputum smear positive or had comorbid conditions; and an ambulatory model for patients who were otherwise well and could be treated in their community.

When introduced in 2011, the policy was not accompanied by dedicated or ring-fenced funding, except for limited support for building and infrastructure through the Global Fund. Provincial and district health officials noted that the policy was ‘an unfunded mandate’; some resorted to funding additional staff and equipment through their general health budgets as well as through the national grant ear-marked for HIV (Jassat, 2020).

Essential elements to assess readiness of a site to provide decentralized DR-TB care included access to laboratory services for diagnosis of DR-TB, uninterrupted supplies of TB drugs and audiology services (Department of Health and Republic of South Africa, 2019). Beyond the ‘hardware’ necessary to deliver services, prerequisites included ‘software’ components such as functionality of multidisciplinary teams, integration of DR-TB care within PHC services, good communication across levels of the system, and effective advocacy and social mobilisation in the community (Appendix 2). These components, indicative of effective management of organizational change processes, could not be assumed to be uniformly present across district health systems in the country.

Key informants consistently pointed to gaps in readiness to implement the policy. These included a lack of sufficient evidence, insufficient resources and time and the absence of concrete plans (Moshabela *et al.*, 2020). Underpinning these considerations was the wider perception of tuberculosis as a stagnant field, slow to change its ways. One senior NGO representative noted that the prospect of decentralising TB was ‘going against tradition’; those who managed TB were ‘very set in their ways…a real old boys’ club’. This key informant further elaborated: ‘There was no expert opinion…it was just that we have this back log, people are dying, what strategy can we employ?’ adding that ‘…as much as a researcher and a scientist you want the evidence before you can implement new things, we didn’t have that luxury’. In EC, one clinical manager referred to the decentralization policy framework as a ‘zero plan’ recalling that ‘we just did what we thought’.

Practical implementation of a decentralized DR-TB service required that patients who had previously been admitted for specialised treatment in a highly monitored environment would now be initiated on to treatment and monitored at district-level units, which traditionally did not handle such complex conditions. The centralized, provincial Centre of Excellence was to remain responsible for initiation and treatment monitoring for XDR-TB patients and other complications. Following treatment initiation, patients would be referred to their nearest primary health care facility for daily observed treatment (DOT), injections, and monitoring of side effects and adherence (Vanleeuw *et al.*, 2020). However, limited funding, inadequate infrastructure, differences in systems capacity (Cox *et al.*, 2017b) and the absence of operational guidelines for implementation left the policy open to interpretation, leading to adaptive responses to compensate and ‘buffer’ the additional clinical load and scope of work placed on systems. In the following sections, we highlight health systems actors’ ability to cope, adapt and devise locally relevant strategies to deliver decentralized care against the backdrop of limited systems ‘readiness’. This was manifest in four key areas: first, re-organization of service delivery; second, redressal of resource shortages; third, treatment adherence support systems; and fourth, extension of care parameters for vulnerable patients.

### Re-organization of service delivery

Getting patients on to treatment as soon as possible is deemed ‘…one of the pillars of success in TB management’ (HAST co-ordinator, female, white, rural WC). To improve access and timely initiation of DR-TB treatment at the district level, services were re-configured:

*Out of the sites that we had identified as our outreach points, we changed the order of things to accommodate seeing the* [DR-TB] *patients there* (…) *it sort of became like a motto that they need to ensure that the space is on a certain day that you don’t have a clinic for instance, like an under-5 for instance, or you don’t have an ANC on the day* (Clinician, female, coloured, rural clinic, WC).

In some rural settings, clinics only had a medical officer in attendance once a week, so arrangements were made to accommodate growing numbers of DR-TB patients. For example, in WC, staff from a rural clinic drove out to satellite clinics to reach farm workers as there were no nearby sites initiating treatment. In KZN, clustering arrangements were made to accommodate patients with poor access to clinics:

*Patients can’t even get to any clinic* (…) *So they know that they are seeing this cluster of clinics, they know the doctor will be there the first day of the month so on that day the patient will be reviewed by the doctor. The patient will have collection of sputum, collection of bloods*, [they will] *do everything on that day, because it’s the only time they get access to the health services* (Clinical manager, male, coloured, urban TB hospital, KZN).

In WC, senior clinicians’ close, cohesive relationship with hospitals in their districts allowed them to manipulate resources to optimize patient care, e.g. moving patients between different facilities to accommodate newer, sicker patients in exchange for stable, recuperating patients.

Rural clinics without frequent access to a clinician devised a system of ‘virtual consultations’ through remote faxing of prescriptions. In rural areas of WC, this involved sending results through to the Infectious Diseases hospital, with the attending physician faxing back the prescription. In other instances, nurses went out of their way to ensure timely access to treatment, e.g. taking sputum samples to the lab personally in order to obtain a diagnostic result quickly.

### Redressal of resource shortages

In developing strategies to deliver decentralized care for DR-TB patients, clinic staff had to absorb existing or anticipated resource gaps. Staff, infrastructure, equipment and drugs were temporarily transferred within and across sites to ensure that patients could access services without interruption.

#### Staff

In areas with limited trained staff or staffing shortages, interventions to deliver the minimum of care were achieved through task shifting and sharing. One rural WC clinic lacking a clinician trained in DR-TB was visited monthly by a doctor from a neighbouring facility who wrote out 6-month prescriptions to be facilitated by the pharmacy and the attending TB nurse.

Outreach services were organized to extend DR-TB expertise to rural and underserved areas. In KZN, the manager (male, coloured) of a specialized TB hospital commented: ‘I think it is a good compromise… shifting the staff to the area of need since we are still getting the patients coming in as outpatients…it is even nicer to know that the doctor is there and the nurses are also there’.

Recognition of systems weaknesses and gaps in specialized care further led to referrals to enable better care pathways for patients who required specific services, such as surgery, mental health resources or rehabilitation due to more complicated forms of DR-TB.

#### Infrastructure and equipment

Infrastructural changes were made to accommodate patients, e.g. an old canteen area for staff was converted into a four-bed DR-TB ward in rural EC. In another rural hospital in EC, staff created their own NGO to provide transport funding for patients unable to access a hospital ambulance. When essential equipment recommended to monitor the effects of DR-TB drugs was not functional or readily available, adaptive solutions were found:

*There was a time that our ECG* [electrocardiogram] *was faulty, so we just went to a nearby clinic …I would try to get patients to come on one specific day if they needed an ECG and then one of SPN’s* [Senior Professional Nurse] *would go and collect the machine* [in her car] *from the other clinic and then I would do all of them* (DR-TB nurse, female, coloured, urban clinic, WC).

*I don't have my own machine. Currently, I'm using one of trauma’s machines in my room* (DR-TB nurse, female, coloured, urban Community Health Centre (CHC), WC).

In some settings, addressing the infrastructural and equipment gaps included leveraging resources through other programmes:

*The equipment and the services might not always be on site but we have been able to access those … with the whole Ideal Clinic*^2^*and all of those other things coming up, it has also been an opportunity to motivate for additional equipment* (Operational manager, female, coloured, urban clinic, WC).

#### Drugs

Successful TB management relies on a steady supply of drugs. Clinic staff anticipated drug stock-outs by ordering larger quantities of drugs in advance or by balancing stock levels across facilities:

*We know that for TB, HIV and certain chronic medical conditions you should always try and make sure that you always order at least 3 months’ worth of stock, every month (…) because from time to time there are drug shortages in the country* (Pharmacist, female, black, rural hospital, EC).

*From my side, I will find out if it* [drug stockout] *is only here or if it is a problem from our pharmacy’s side or it if it is out of stock in general from the depot … then I will speak to the pharmacist in charge and she will contact other clinics to ask what their stock levels are. If we are really short, we will do our utmost to go out to other clinics to get some stock to cover* (DR-TB nurse, female, coloured, urban clinic, WC).

### Creation of treatment adherence support systems

The strict monitoring demands of decentralized delivery of complex drugs in ambulatory settings obliged HCWs to adopt flexible practices related to drug dispensing, keeping patients on treatment, documentation practices and sharing of expertise.

#### Drug dispensing

Clinicians described adaptive prescribing practices to support patients on treatment, e.g. tapping into the existing packaging and delivery services for the patient’s monthly anti-retroviral (ARV) drug provision or giving patients supplies of treatment ‘under the table’ when patients were unable to come back on a daily basis. Patient costs incurred through multiple visits were also a reason to offer a more flexible schedule of treatment:

*Most of our MDR paeds* [paediatric cases] *will have to pay up to two hundred rand to get to the hospital… you know four hundred rand* [27 US$] *for a return trip for the mother and child or children where there are two or three of them on treatment is tricky. So, I often give them two months of treatment at a time* (Hospital clinician, female, white, rural hospital, EC).

In order to establish and maintain treatment routines, HCW adopted common-sense modifications of existing prescribing practices to facilitate dispensing of medication. These included pre-packing, bulk preparation and colour-coding medicines:

*We prepack the injections and then I literally write thirty scripts effectively and they have to sign on each one every day* (Provincial hospital doctor, male, white, EC).

*I was using the medicine containers, the small ones in which they decant ointment in… I have a small booklet where I will put the sticker of who is coming tomorrow then I will say ok this one* [this patient], *I can give* [medicines] *weekly* (DR-TB nurse, female, coloured, urban clinic, WC).

*You find, like in one household there’s three kids. So, what we usually do with those is, in terms of dispensing, we colour-code the medication* (Pharmacist, female, black, rural district hospital, EC).

#### Keeping patients on treatment

To reduce the frequency of visits, HCW assessed adjusted treatment schedules based on individual patients’ situations. For example, nurses reported giving treatment on a weekly basis to patients who were working, no longer infectious, or deemed stable in terms of their treatment adherence. Strategies were devised for patients unable to attend a daily clinic, sometimes enlisting the help of other patients to collect treatment or an NGO to provide DOT at home:

*Even those who have gone back to work we organise a system for them. I don't know but with this patient now currently there are always two that are staying near to each other and then this one is going to work and the other one will come fetch his treatment and give it to him* (Operational Manager, female, coloured, urban CHC, WC).

*The patients prefer to be at home and we also prefer them to be at home. So, we would involve our NGOs* (…) *and they would DOT* [directly observe treatment] *them at home. The patient would still have to come once a month to see myself and the sisters for the investigations and the clinical examinations* (Family physician, female, white, rural Community Day Centre (CDC), WC).

Clinic staff described numerous ways of motivating patients to stay on treatment. Some organized adherence workshops to ‘boost’ patient morale. The operational manager of an urban CHC in WC spoke of maintaining ‘open communication’ with patients, e.g. through WhatsApp. One DR-TB nurse, also in an urban WC clinic celebrated treatment ‘successes’ by organizing parties and treatment completion ‘certificates’ that had been designed by the facility co-ordinator.

When patients needed to be in in-patient care longer than recommended, e.g. because of their specific difficulties to stay on treatment, HCW found ways to extend the prescribed length of stay.

*At times you get patients that request the admission for a little bit longer than actually clinically needed and very often they say the temptations out in the community are just too bad and they know that they’re going to have difficulty managing it* [adherence] (Doctor, female, white, rural hospital, WC).

Referring to patients who had to return to work soon after they were no longer considered infectious and their government TB disability grant had run out, one DR-TB nurse (female, coloured) in an urban WC clinic commented: ‘Occasionally, you have to sort of override the protocol a bit if you want to keep the patient in care’, later adding that ‘their bosses don’t always understand’ the long-term debilitating effects of the illness and of being on treatment.

#### Documentation

‘Tinkering’ was also evident in initiatives to facilitate record-keeping and support the complex monitoring and documentation needs of decentralized DR-TB treatment. Monitoring forms that were seen to be cumbersome were re-designed to make them more user-friendly. In part, these modifications were aimed at reducing HCW unfamiliarity with new protocols and processes that were introduced as a result of the mandate to decentralize care:

*At the beginning it is not that easy you know for somebody that sees them* [the patients]*. Often you know what to do but even the medicines and the names were completely new. So, I developed like a worksheet that will tell you or guide to do sputum monthly, it will guide you to how often you need the ALT, how often you need the blood tests, how often audiology, how often X-rays so if a doctor went according to the worksheet, you couldn't miss something* (Clinician, female, coloured, rural district CDC, WC).

*There are certain things that I have designed to make it easier for them* [HCW] *to work, like I redesigned the monitoring tool of the drug-resistant TB. So, we have a shorter one and a longer conventional one that I have designed* (Sub-district co-ordinator, female, coloured, urban clinic, WC).

#### Sharing expertise

Some clinicians developed paper-based registers and templates to identify problems for discussion on a monthly basis, enabling timely and ongoing in-service training. Existing gaps in expertise were also addressed through support and mentorship networks among health professionals, often using WhatsApp as a platform. These served to discuss difficult cases, disseminate information and access experts. In a rural district hospital in EC, the senior medical officer managed DR-TB patients in collaboration with an off-site specialist consultant.

### Extending parameters of care for vulnerable patients

Beyond meeting basic requirements, some staff actively sought to address the social needs of patients who were impoverished or had difficult life circumstances. Nurses in rural areas of WC reported recording lower patient weights or adjusting scales in order to help patients to get into nutritional support programmes. One nurse in WC described building relationships with ward councillors and community members to organize food parcels for vulnerable patients.

*There was a thing where they said that there were food trolleys … I jumped forward because I know what the needs of the family with five MDRs are. I would write to the ward councillor* [to say] *‘there are so many people in that house and all of them are not getting the grant with the exception of the grandmother and she is also sick’. I give them a hand* (…) *Food packages that we make up on our own are accepted at certain NGOs to help them* (Nurse, female, black, rural clinic, WC).

Clinic staff pro-actively intervened to mobilize community assistance for patients living in sub-standard housing.

*There was this family, a sister was living with her two brothers, the mother passed away and then this boy contracted TB when he was 16 years of age and he was diagnosed with HIV* (…) *they were living in a one room shack then I had to intervene while we were still waiting for a bed. I had to ask the community to get involved because there was no material to find if whoever can then make a shack for him* (Clinic manager, female, black, rural clinic, WC).

*I will go and have a look and see where you* [referring to a patient] *live. I link up with the ward councillor. I link up with housing and I will go and look at what the problem is there and then I will talk to them*. [I will say :] *‘There are so many people in the house…can’t you add a bungalow to it or can you give me a hand?’* (Nurse, female, black, rural hospital, WC).

Finally, initiatives to cater to children’s needs were observed, e.g. in a rural hospital in EC, where ‘individual plans’ were made in order to accommodate mothers and children together. In this hospital, staff also supported out-of-school children during their treatment:

*We’ve helped them* [kids] *with school and stuff as well. Our OTs* [occupational therapists] *will go and give them extra lessons or our social worker will help get them into the local school for two months which helps a lot because for children that is the most important thing* (Senior Medical Officer, female, white, rural hospital, EC).

## Discussion

Recognition of the importance of strengthening health systems capacity and readiness to deliver priority interventions has increased over the past 15 years, particularly in low- and middle-income countries (LMIC). For the most part, tools to assess ‘readiness’ for uptake of specific policies or scale-up of existing interventions in LMIC involve mapping essential resources, knowledge and skills needed to implement a new intervention or initiative at facility-level (WHO 2015). While useful in ranking facilities according to their capacity, in principle, to provide basic health services at minimum standards (Jigjidsuren *et al.*, 2019; Ssensamba *et al.*, 2019), these tools lack consideration of the software dimensions of ‘readiness’ related to individual and collective agency to implement change.

Consequently, a few researchers have begun to articulate their own fit-for-purpose ‘systems readiness frameworks’. Reporting on a novel framework for assessing readiness to implement a domestic violence intervention at primary care level in Palestine, Colombini *et al.* (2020) conclude that even if all the necessary ‘hardware’ elements are in place, ‘…the materialization of collective readiness is dependent on the software elements also being ready’. Conversely, Akinyemi *et al.* (2019) discuss how, in the absence of adequate ‘hardware’ for the scale-up of community-based distribution of injectable contraceptives in Northern Nigeria, health workers enable policy implementation through their adaptive responses: ‘…they often modify the process in order to adapt to the realities on the ground’.

Our study of health systems actors’ emerging responses to the policy of decentralizing DR-TB care in South Africa suggests there are useful bridges to be made across the currently distinct bodies of literature on health systems and organizational readiness. We concur with May *et al.* (2016) that understanding organizational aspects of implementation requires attention to how ‘they are shaped by the behaviours and actions of participants as they negotiate the normative and relational environment in which they are set’. We observed numerous instances of bottom-up ‘tinkering’ that challenge a linear interpretation of policy implementation, reflecting actors’ resilience in managing everyday ‘micro-level crises’ (Barasa *et al.*, 2017, 2018) but also their capacity for managing change.

Observed practices contributed to strengthening different capacities of resilient systems, described in the development literature as absorptive, adaptive and transformative. For the most part, HCWs and managers strived to maintain functional services in the face of policy change. Under *absorptive* capacity, we noted actions that sought to restore balance in observed disparities in resource allocation and capacity across sub-components of the system. *Adaptive* capacity was evident in HCWs’ refinement of existing tools and practices and their extension of tasks to accommodate patients’ unique and challenging circumstances. Less frequently, HCW and managers’ actions demonstrated *transformative* capacity in their attempts to organize additional or novel ways of facilitating patient access, care and follow-up.

‘Tinkering’ may thus serve different purposes in this setting: the absence of operational guidelines for policy implementation may open the space for ‘tinkering’ that is undertaken to meet minimum requirements for a functional delivery system. In other instances, however, health systems actors’ ‘tinkering’ extends beyond the status quo, demonstrating readiness to implement change towards improving quality of care (Mussie *et al.*, 2020). Relevant to this distinction are the kinds of organizational cultures that support adaptive practices in the clinic environment (Weiner, 2009); HCWs’ capacity to provide individualized care may have less to do with available resources or the policy architecture, than with its ‘soft periphery’ (Langley and Denis, 2011) that allows for discretionary decision-making space and power within specific contexts.

Our focus is on decentralized DR-TB care in South Africa, yet health systems actors’ ‘tinkering’ occurs in most settings where new service delivery initiatives are introduced. Studying the ‘micro-politics’ of implementing interventions to improve health care delivery (Langley and Denis, 2011) is a relatively recent turn in high-income countries, but still rare in studies on health systems in LMIC. Most literature on ‘organisational readiness’ stems from high-income settings that do not share the resource constraints and challenges of many LMIC health systems; accordingly, assumptions regarding both individual and collective agency may not apply.

Our study suggests that assessing front-line health workers’ capacity to cope, adapt and innovate within particular organizational contexts may enhance existing tools to assess ‘systems readiness’ for implementing policy initiatives. Currently, standardized assessments use binary checklists to establish the presence or absence of components needed to deliver a service. Relatively simple adjustments to both tool and method of application would enable assessment of organizational and individual capacity to withstand negative shocks (resilience) and be prepared for change (readiness). A limited set of open-ended questions or vignette scenarios might be added to assess when and why resource gaps occur, how they compromise service delivery, and what HCWs and managers do to address these situations. In addition to understanding how health systems actors ‘get by’ and cope with what they have, it is important to document instances where they go ‘over and beyond’ what is required to provide patient-centred care. While these instances may partly reflect the social fabric of health facilities, they conversely may also signal the potential for stress and exploitation in chronically under-resourced settings.

### Study strengths and limitations

Our study draws on a large data set collected over 2 years by researchers with extensive familiarity with the changing landscape of TB and DR-TB care and its delivery. Triangulation of methods, discussions among research team members following each phase of data collection, and iterative consultations with staff at research sites increased trustworthiness of the data obtained. We focused on three out of nine provinces in South Africa and did not collect data from private health care facilities; furthermore, data on which this paper is based stems mainly from our interviews in WC and KZN. However, earlier research and the insights gained from Phase 1 (Cox *et al*. 2017a; Moshabela *et al* 2020) provide evidence that the practices observed across urban and rural facilities in these two provinces were fairly uniform and representative of early responses to the policy across the country. We note, however, that the majority of our cited examples stem from WC. This is likely due to the fact that decentralization of DR-TB care was already considerably advanced in WC by the time the national strategy was released in 2011 as compared with KZN, for example (Vanleeuw *et al.*, 2020). WC also has a history of implementing their own policies, as can be seen in the early introduction of decentralized delivery of ARV therapy for HIV (WHO, 2003).

Although we argue that ‘tinkering’ may provide clues as to why some systems are more ‘ready’ to implement policy than others despite resource gaps, the study this paper draws on did not explicitly set out to compare early vs delayed implementation of policy in the districts studied. Furthermore, while our study focused on positive practices supporting decentralized DR-TB care, we are aware that these may be difficult to sustain, creating an unacceptable burden for some HCW who have to ‘make do’ with inadequate resources or support. HCW ‘tinkering’ may also have detrimental consequences for patient care (Mwamba *et al.*, 2018). For example, dispensing medicines to ambulatory DR-TB patients for lengthier time periods may mean HCWs miss the opportunity to monitor side effects, compromising the quality of care provided.

## Conclusion

In a quickly moving landscape of policy, funding and technological developments in DR-TB care in South Africa, HCWs and managers responded to the policy initiative to decentralize DR-TB care through small acts of ‘tinkering’ as well as more deliberate strategies to deliver sustained services. Our focus on ‘tinkering’ illustrates some of ‘the things that people do to make something happen’ (May, 2013) in the implementation of complex interventions. A bottom-up examination of these practices can shed light on the conditions that generate variability in interpretation and ‘successful’ implementation of policy directives, but also raise moral questions about placing accountability for policy implementation on HCW operating in sub-optimal conditions.

Our observations support the need to develop actor-oriented frameworks of health systems ‘readiness.’ Currently, piloting of a ‘harmonised approach’ to health facility assessments that intends to overcome the ‘piecemeal’ focus on specific service areas is underway (WHO, 2019), a promising, but limited move in our view. Advancing the field of health systems ‘readiness’ assessment will require more radical revision to include real-time capture of human capacities not only to mitigate systems constraints, but to drive systems change. For TB services in South Africa and elsewhere, acute gaps between rhetoric and reality of people-centred care (Odone *et al.*, 2018; Furin *et al.*, 2020) suggest that close attention to the conditions that promote adaptive capacity as well as the emergence of ‘change agents’ is critical.

*Conflict of interest statement*. None declared.

## Funding

The work presented in this paper was supported by the Joint Health Systems Research Initiative, jointly supported by the Department for International Development (DFID), the Economic and Social Research Council (ESRC), the Medical Research Council (MRC) and the Wellcome Trust [grant number MR/N015924/1]. This UK funded award is part of the EDCTP2 programme supported by the European Union. Ethical approval for the project was obtained through the University of Cape Town Human Research Ethics Committee (HREC REF 350/2016). HC is supported by a Wellcome Trust Fellowship.

## Endnotes

1 A rapid automated diagnostic test called Xpert MTB/RIF that could detect resistance to rifampicin, a key drug used to treat tuberculosis, was introduced between 2011 and 2013, resulting in an increase in the number of individuals identified with rifampicin-resistant TB. In 2014, bedaquiline—a drug with considerably less toxic side-effects than standard regimen—was registered for ‘compassionate use’ in South Africa; thousands of individuals received the drug through the expanded access programme until it was more widely released in 2018 (Ndjeka *et al.*, 2015).

2 The Ideal Clinic programme, launched in South Africa in July 2013, intended to systematically improve the quality of care provided in Primary Health Care facilities.

## Appendix 1

### List of themes

**Table:**

Key theme	Sub-themes
Systems readiness gaps	DR-TB policy vs practice discursive gaps
	Lack of operational guidelines
	Lack of funding
Adaptive capacity	Re-organizing mode of service delivery
	Addressing resource shortages and gaps (drugs, equipment, staff)
Local innovations	Novel communication and knowledge transfer mechanisms
	Monitoring and motivating patients on treatment
	Extending job/care parameters for specific populations

## Appendix 2

*Source:* Department of Health, Republic of South Africa (2019, p. 17).
